# ^99m^Tc-rituximab tracer injection for guiding internal mammary sentinel lymph nodes biopsy in primary breast cancer: A prospective observational study

**DOI:** 10.3389/fonc.2023.1100077

**Published:** 2023-02-10

**Authors:** Wenxin Chen, Yaodong Su, Hui Zhang, Yu Zhang, Lin Zhu, Mengbo Lin, Zhiyi Lin, Mingdian Yu, Shengping Yang, Yanmin Zhang

**Affiliations:** ^1^ Department of Nuclear Medicine, Shengli Clinical Medical College of Fujian Medical University, Fuzhou, China; ^2^ Department of Nuclear Medicine, Fujian Provincial Hospital, Fuzhou, China; ^3^ Fujian Research Institute of Nuclear Medcine, Fuzhou, China; ^4^ Department of Oncology Surgery, Shengli Clinical Medical College of Fujian Medical University, Fuzhou, China; ^5^ Department of Oncology Surgery, Fujian Provincial Hospital, Fuzhou, China; ^6^ Department of Ultrasonic Diagnostics, Shengli Clinical Medical College of Fujian Medical University, Fuzhou, China; ^7^ Department of Ultrasonic Diagnostics, Fujian Provincial Hospital, Fuzhou, China

**Keywords:** breast cancer, ^99m^Tc-rituximab, sentinel lymph node, internal mammary lymph node, single photon emission computed tomography/computed tomography, biopsy

## Abstract

**Objective:**

To explore the use of ^99m^Tc-rituximab tracer injection for internal mammary sentinel lymph node (IM-SLN) detection in patients with primary breast cancer.

**Methods:**

This prospective observational study enrolled female patients with primary breast cancer between September 2017 and June 2022 at Fujian Provincial Hospital. The participants were divided into the peritumoral group (two subcutaneous injection points on the surface of the tumor), two-site group (injections into the glands at 6 and 12 o’clock around the areola area), and four-site group (injections into the gland at 3, 6, 9, and 12 o’clock around the areola area). The outcomes were the detection rates of the IM-SLNs and axillary sentinel lymph nodes (A-SLNs).

**Results:**

Finally, 133 patients were enrolled, including 53 in the peritumoral group, 60 in the two-site group, and 20 in the four-site group. The detection rate of the IM-SLNs in the peritumoral group (9.4% [5/53]) was significantly lower than in the two-site (61.7% [37/60], P<0.001) and four-site (50.0% [10/20], P<0.001) groups. The detection rates of A-SLNs among the three groups were comparable (P=0.436).

**Conclusion:**

The two-site or four-site intra-gland injection of ^99m^Tc-rituximab tracer might achieve a higher detection rate of IM-SLNs and a comparable detection rate of A-SLNs compared with the peritumoral method. The location of the primary focus has no impact on the detection rate of the IM-SLNs.

## 1 Introduction

Breast cancer is the most common cancer in women, and the incidence and mortality rates increase yearly. In 2020, the international agency for research on cancer (IARC) of the World Health Organization (WHO) released the latest global cancer burden data (1): in 2020, there were 2.26 million new breast cancer cases in the world, surpassing lung cancer. Breast cancer incidence is higher in high-income countries (571/100 000) than in low-income counties (95/10 000) (2).

The TNM staging of breast cancer is directly related to the prognosis of the disease, and the staging of the lymph nodes is an important factor (3). According to the NCCN guidelines, patients with negative ipsilateral axillary lymph nodes (ALNs) should still be classified as N2 if an ipsilateral internal mammary lymph node (IMLN) is positive, while patients with ipsilateral positive IMLNs and ALNs are N3 (3). Hence, IMLN metastasis has a prognostic significance independent from the ALN status (4–6). Therefore, the internal mammary sentinel lymph nodes (IM-SLNs) in breast cancer are significant for TMN staging and formulating the treatment regimens, and patients with positive IM-SLNs will be eligible for more aggressive therapies even in the absence of positive axillary sentinel lymph nodes (A-SLNs) (3). Nevertheless, detecting IMLNs is difficult due to the complex anatomical structure around the IMLN chain.

The detection rate of IM-SLNs using the traditional radioactive tracer (^99m^Tc-sulfur) injection method (i.e., two subcutaneous injection points on the surface of the tumor) is low (7). Mudun et al. (8) reported that injecting ^99m^Tc-sulfur colloid into the breast gland could improve the detection rate. Ultrasound-guided intraglandular injection ensures that the tracer can be accurately injected into the breast gland. Qiu et al. (9) showed that the detection rate of IM-SLNs was also related to breast density. Increasing the injection volume can improve the detection rate of IM-SLNs, and the detection rate of IM-SLNs could increase from 13% to 71% using large-volume intraglandular injection (8, 9). The tracer ^99m^Tc-sulfur colloid (SC) mainly accumulates in lymph nodes after the colloid is engulfed by lymphocytes. Then, these lymph nodes can be detected using a γ-detector during the operation. Still, the time window is narrow, and increasing the injection-biopsy delay often leads to developing additional SLNs, resulting in excessive dissection (10).

^99m^Tc-rituximab is a human-mouse chimeric monoclonal antibody targeting the CD20 molecules on the surface of B lymphocytes and can be used to display SLNs (11, 12). ^99m^Tc-rituximab is a lymph node tracer that targets CD20 on the surface of the B lymphocytes found in lymph nodes. It has an important application value in the SLN biopsy (SLNB) of ALN and is superior to the traditional ^99m^Tc-SC and other radioactive tracers (11, 12). Li et al. (13) showed that ^99m^Tc-rituximab has the characteristics of rapid clearance from the injection points, long retention time in the SLNs, good localization performance, and time flexibility of surgery. Compared with traditional tracers, ^99m^Tc-rituximab has advantages, but none of the studies on ^99m^Tc-rituximab involved IM-SLNs.

Therefore, this study aimed to explore the impact of the ^99m^Tc-rituximab tracer injection on IM-SLNs detection in primary breast cancer patients.

## 2 Methods

### 2.1 Study design and patients

This prospective observational study included female patients with breast cancer who underwent surgery between September 2017 and June 2022 at the authors’ hospital. This work was carried out in accordance with the Declaration of Helsinki (2000) of the World Medical Association. This study was reviewed and approved by the ethics committee of the authors’ hospital (K2017-09-069). All patients provided a signed written informed consent form.

The inclusion criteria were 1) breast cancer confirmed by pathology, 2) without distant metastasis, and 3) able and willing to sign the informed consent form. The exclusion criteria were 1) previous history of axillary, internal mammary, or chest surgery on the affected side, 2) inflammatory breast cancer, 3) patients with lymph node metastasis in the inner mammary region found by imaging examination, 4) pregnancy or lactating, or 5) patients who accepted neoadjuvant chemotherapy.

### 2.2 Procedure

Rituximab (Rituxan) was purchased from Roche. The labeling protocol was conducted as previously reported (13). ^99m^TcO4- was provided by Guangdong CI Pharmaceutical Co., Ltd., Fuzhou Branch. The tracer’s radiochemical purity (RP) (^99m^Tc-rituximab) was always greater than 97% when tested by radio-TLC and HPLC. A GE Discovery NM/CT 670 pro dual-head single photon emission computed tomography (SPECT)/computed tomography (CT) was used for preoperative imaging. A Neo 2000™ γ-detector (Johnson & Johnson, USA) was used to detect the lymph nodes during the operation.

**Figure 1:**
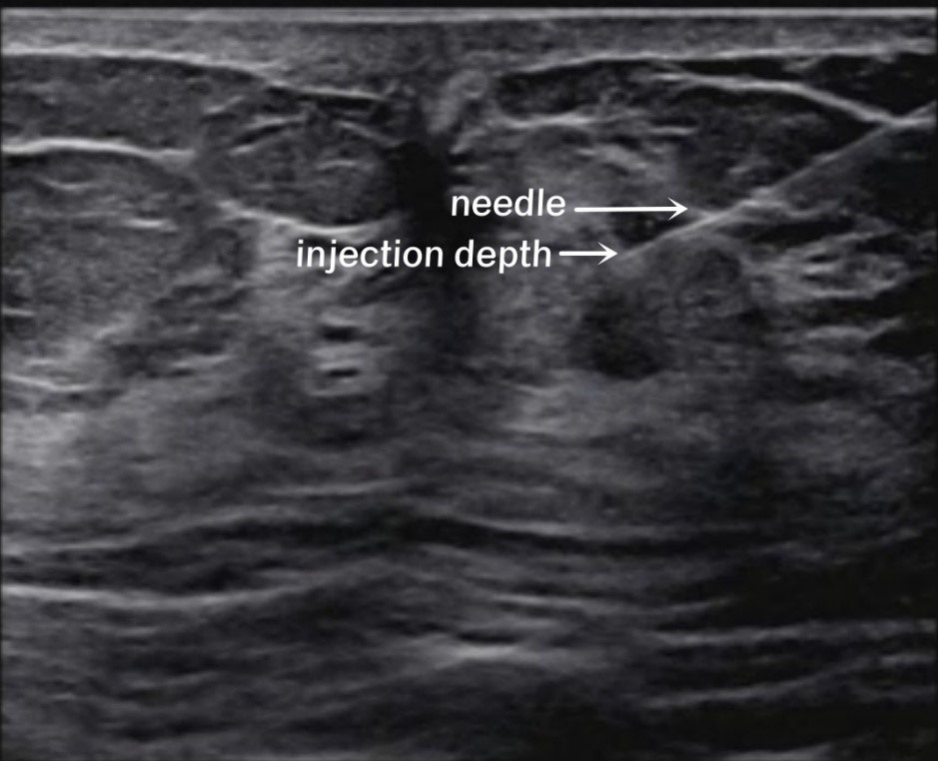
Ultrasound-guided intraglandular injection around the areola. The white arrows show the needle and the injection depth (6 o’clock).

In this study, the researchers explained each type of injection to the patients, including the possible advantages and disadvantages, and the patients decided which kind of injection they wished to receive. The patients’ condition did not affect their choice. The patients were divided into three groups based on the injection method: peritumoral, two-site, and four-site. In the peritumoral group, ^99m^Tc-rituximab was injected at two subcutaneous injection sites on the surface of the mass 2-4 h before the operation. In the two-site group, ^99m^Tc-rituximab was injected into the breast parenchymal gland under ultrasound guidance 2-4 h before the operation (**Figure 1**), and the 6 and 12 o’clock positions around the areola area were selected as the injection sites, 1 cm away from the nipple. In the four-site group, ^99m^Tc-rituximab was injected into the breast parenchymal gland 2-4 h before the operation under ultrasound guidance, and the 3, 6, 9, and 12 o’clock positions around the areola area were selected as the four injection points, 1 cm away from the nipple. The total injection volume of each injection site was 1 ml. A total of 17.5-29.6 MBq was injected, and SPECT/CT imaging was performed 1–3h (Correction of clerical errors) later.

All patients underwent preoperative lymphoscintigraphy. The patients were placed in the supine position, their upper limbs were lifted, the elbows were placed on the forehead, and the armpits were fully exposed. The injection site was placed in the middle and lower part of the field of vision, and fusion tomography was performed. The radioactive concentration outside the injection points was defined as SLN. A total of 60 frames (6°/frame) were collected by SPECT/CT, and the acquisition time of each frame was 10 s. The acquisition conditions were tube voltage at 120 kV, tube current (usually 80 mA) was changed according to the acquisition position and the degree of obesity and thinness of the patient, and scanning layer thickness of 10 mm. The collected data were reconstructed by OSEM (reconstruction layer thickness of 1.25 mm), and the CT, SPECT, and SPECT/CT fusion images of the cross-sectional, coronal, and sagittal planes were obtained. The γ-detector was used to detect the “hot spot” to determine the SLN. The “hot spot” was determined compared with the background radioactivity count of 2:1-3:1 before resection and 10:1 after resection (14, 15). The resected SLNs were sent for rapid frozen pathological examination. The detection rate of IM-SLNs (%) was determined by dividing the pathological diagnosis of SLNB into IM-SLN by the percentage of total cases. Similarly, the detection rate of ALN was calculated. IM-SLN was determined by pathology and calculated as true positive.

### 2.3 Outcomes

The primary outcome of the study was the detection rate of IM-SLN. The secondary outcome was the detection rate of ALN. The detection rate of IM-SLN (%) = imaging and the number of IM-SLN cases detected by the γ-detector/total number of cases confirmed as IM-SLN by SLNB pathology × 100. The detection rate of ALN (%) = imaging and the number of A-SLN cases detected by the γ-detector/the total number of A-SLN cases confirmed by SLNB pathology × 100.

The demographic information (age) and clinical characteristics (including mass location, quadrant distribution, pathological type, and T stage) were also recorded.

### 2.4 Sample size

In this prospective study, the power was 95%, and the α was 0.05. According to the relevant study results of global IM-SLNB, the detection rate of IM-SLN was expected to be 15% in the peritumoral group and more than 65% in the two- and four-site groups (16, 17). According to the calculation using PASS15, at least 19 patients in each group were needed.

### 2.5 Statistical analysis

SPSS 23.0 (IBM, Armonk, NY, USA) was used for statistical analysis. The continuous variables that conformed to the normal distribution were expressed as means ± standard deviation, and the variables with a skewed distribution were expressed as median (range). ANOVA analysis was used to compare the differences among three groups. Categorical variables were expressed as n (%). The chi-square test or Fisher exact probability method was used to compare the baseline data and three injection methods’ detection rates of IM-SLN and A-SLN. Two-sided P<0.05 was considered statistically significant.

## 3 Results

**Figure 2:**
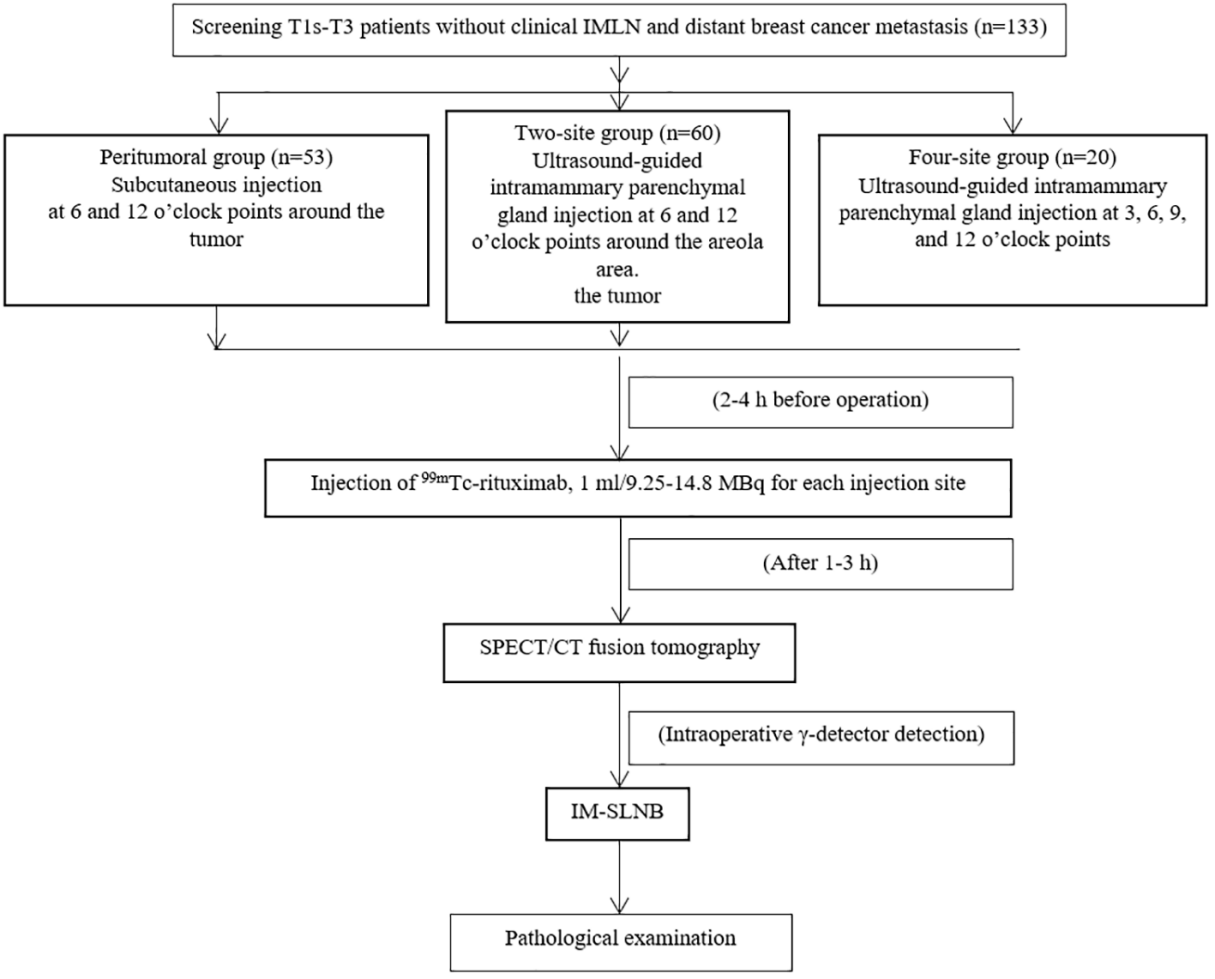
Sentinel lymph node examination process for breast cancer.

This study enrolled 133 patients: 53 in the peritumoral group, 60 in the two-site group, and 20 in the four-site group (**Table 1** and **Figure 2**). The patients were 25-77 years old, with an average age of 51.9 ± 11.1 years and a median age of 52. There were 61 cases of left breast cancer and 72 of right breast cancer. There were no differences among the three groups regarding age, BMI, quadrant distribution, pathological type, T stage, and total injected dose (all P>0.05).

**Table 1:** Characteristics **of** the patients.

	Two-site (n=60)	Four-site (n=20)	Peritumoral (n=53)	P
Age	51.13 ± 11.26	54.90 ± 11.11	51.57 ± 10.99	0.412
BMI	23.54 ± 2.57	23.58 ± 2.69	23.12 ± 2.58	0.644
Quadrant distribution				
Outer upper quadrant	27	7	19	0.455
Outer lower quadrant	6	2	4	
Inner upper quadrant	9	4	5	
Inner lower quadrant	5	0	1	
Center (below the nipple and areola)	2	1	2	
Inner quadrant (9 o’clock on the left and 3 o’clock on the right)	1	0	2	
Outer quadrant (3 o’clock on the left and 9 o’clock on the right)	4	1	6	
Upper quadrant (12 o’clock)	6	3	12	
Lower quadrant (6 o’clock)	0	2	2	
Pathological type				
Invasive carcinoma	49	16	45	0.849
Carcinoma in situ	11	4	8	
T stage				
Tis (carcinoma in situ)	14	3	6	0.780
T1	14	6	15	
T2	24	8	25	
T3	8	3	7	
Total injected dose				
18.5 MBq (0.5 mCi)	10	6	4	0.144
25.9 MBq (0.7 mCi)	25	6	20	
29.6 MBq (0.8 mCi)	25	8	29	

**Figure 3:**
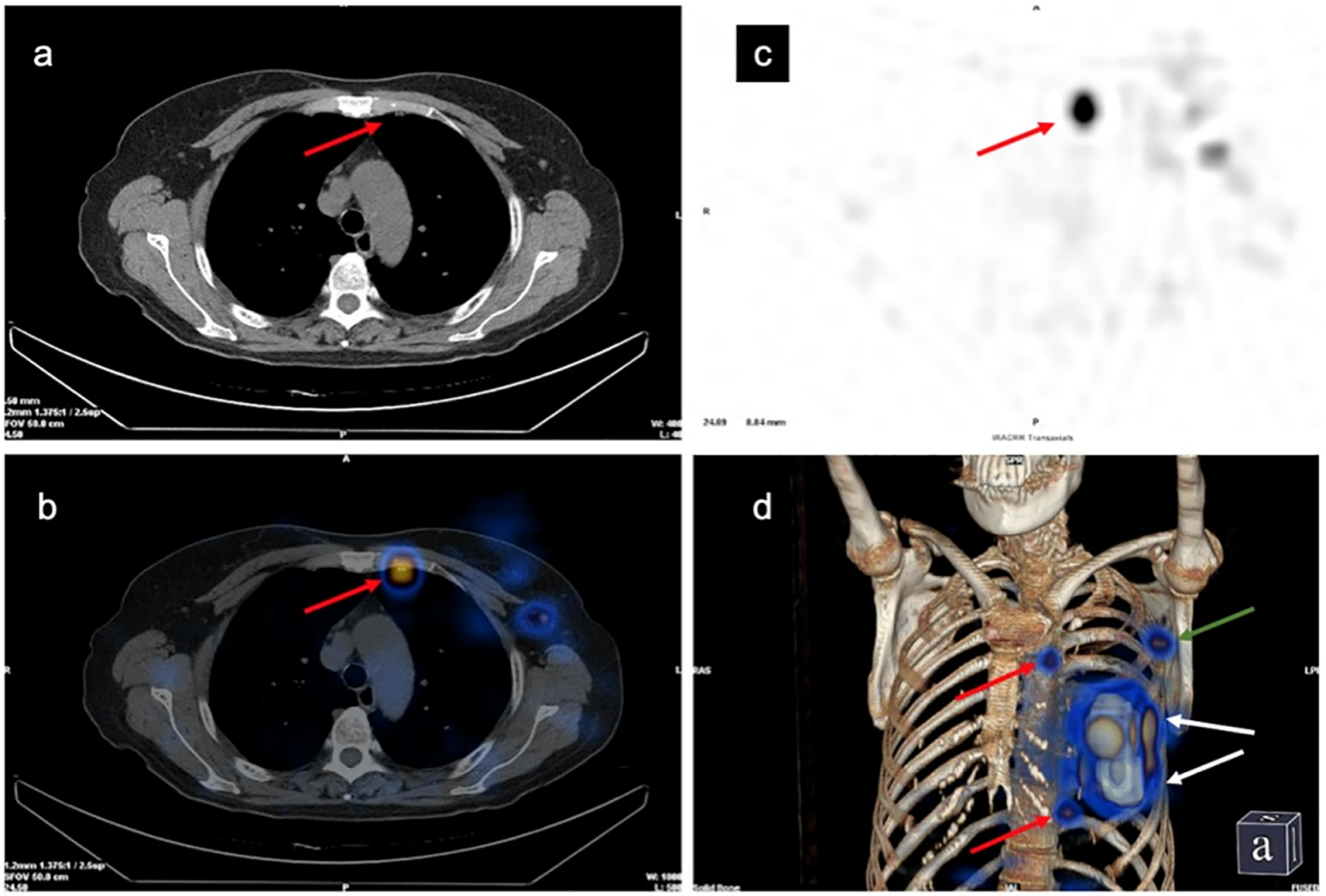
Development of the left internal mammary sentinel lymph nodes (IM-SLN) and left axillary sentinel lymph node (A-SLN). **(A)** Computed tomography (CT) plain scan. **(B)** Single-photon emission computed tomography imaging. **(C)** Tomographic fusion image. **(D)** 3D image. Red arrows, IM-SLNs; white arrows, injection points; green arrow, A-SLN.

**Figure 4:**
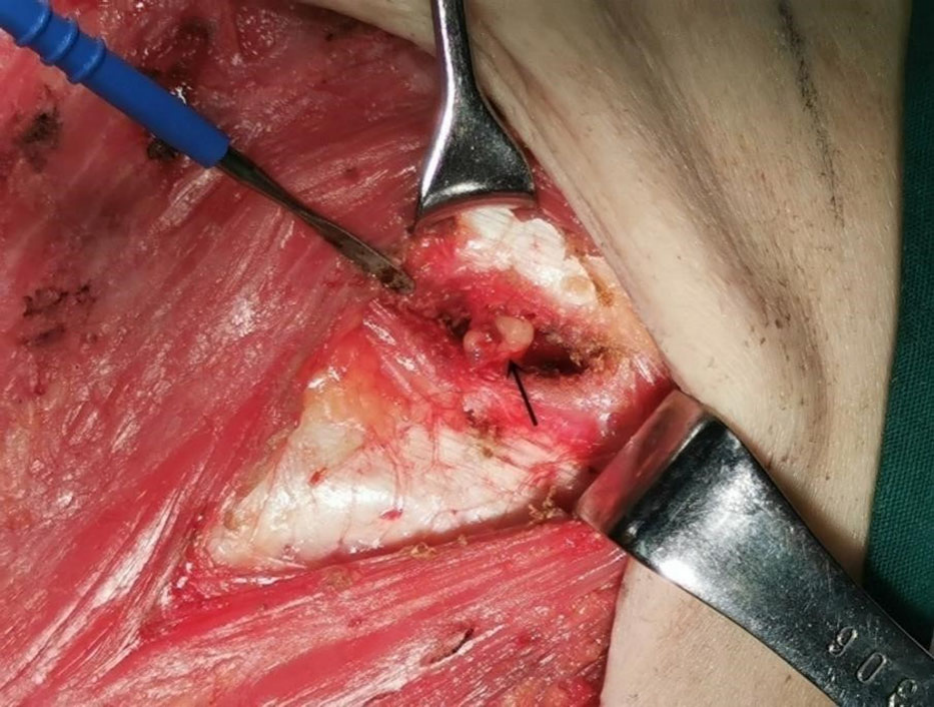
Intraoperative internal mammary sentinel lymph nodes (IM-SLN). The arrow shows the IM-SLN.

SPECT/CT imaging could clearly show the radioactive concentration foci and corresponding lymph nodes (**Figure 3**). The IMLN were small and often undetectable by CT alone, but they were detectable by SPECT. Therefore, imaging focus and intraoperative γ-detector could be used to detect the SLNs (**Figure 4**). The detection rates of the IM-SLNs were 9.4% (5/53) in the peritumoral group, 61.7% in the two-site group (37/60), and 50% in the four-site group (10/20) (P<0.001); the rates were significantly higher in the two-site (P<0.001) and four-site (P<0.001) groups compared with the peritumoral group, but there were no differences between the two- and four-site groups (P>0.05). There were no significant differences in the detection rates of the A-SLNs among the peritumoral (96.2%), two-site (100%), and four-site (100%, P>0.05) groups (**Table 2**). The location of the primary focus had no impact on the detection rate of the IM-SLNs (P=0.943) (**Table 3**).

**Table 2:** Comparison of the detection rates of IM-SLN and A-SLN with different injection methods.

Characteristics	Two-site (n=60)	Four-site (n=20)	Peritumoral (n=53)	P
IM-SLN				<0.001
Positive	37 (61.7)a	10 (50.0)a	5 (9.4)	
Negative	23 (33.3)	10 (50.0)	48 (90.6)	
A-SLN				0.436
Positive	60 (100)	20 (100)	51 (96.2)	
Negative	0	0	2 (3.8)	

a : P<0.05 compared to the peritumoral group.

IM, internal mammary; SLN, sentinel lymph node; A, axillary.

**Table 3:** Relationship between the detection rate of IM-SLN and the location of primary focus in the patients with intra-gland injection of ^99m^Tc-rituximab (n=73).

Characteristic	Outside (n=44)	Inside (n=17)	Upper quadrant (n=12)	P
IM-SLN				0.943
Positive	25 (56.8)	11 (64.7)	7 (58.3)	
Negative	19 (43.2)	6 (35.3)	5 (41.7)	

Outside, outer upper quadrant + outer lower quadrant + outer quadrant; Inside, inner upper quadrant + inner lower quadrant + inner quadrant; Upper quadrant, 12 o’clock.

IM, internal mammary; SLN, sentinel lymph node.

## 4 Discussion

The present study suggests that the detection rate of IM-SLNs using ^99m^Tc-rituximab might be higher when injecting using the two- or four-site method. This study might provide an optional strategy for IM-SLNs screening before invasive lymph node biopsy in the inner mammary region.

In the breast lymph drainage system, 75% of the lymph is drained to the ALNs, and about 25% of the lymph is drained to the IMLNs (18). Cong et al. (19) injected different SLN tracers into different breast positions and finally located the same IM-SLN. The drainage pathways between various parts significantly correlate and are consistent (19). They put forward the view that IM-SLN is not only the drainage pathway of the primary tumor but also the SLN of different breast regions (19). Cao et al. (20) used dynamic lymphoscintigraphy to observe the migration of a small-particle radiotracer (^99m^Tc-Dextran 40) after injection and found that the IMLNs have the same phenomenon of gradual drainage as ALNs (i.e., the lymph is first drained into the IM-SLN, and then to the following IMLNs), confirming that IMLNs can be SLNs of different breast areas, as can be ALNs. Bi et al. (21) also verified this hypothesis through a prospective study. Therefore, in the same way as the A-SLNs, the pathological status of the IM-SLNs can provide important prognostic information for patients with breast cancer. Qiu et al. (22, 23) analyzed the results of IM-SLNB in clinically ALN-negative patients and revealed that the metastasis rate of IM-SLN was only 8.1%. The meta-analysis conducted by Gong et al. (16) suggested that the risk of IMLN involvement was significantly increased in patients with ALN metastasis (OR=6.01, 95% CI: 3.49-10.34). Cong et al. (24) proposed obtaining the histological diagnosis of IMLN through IM-SLNB, which could lead to a more personalized radiotherapy strategy. With the survival benefits of internal mammary node radiotherapy (IMNI) (25–27), the diagnosis and treatment of IMLN have attracted much attention in recent years. Nevertheless, the NCCN guidelines suggest that patients with IMLN metastasis confirmed pathologically should be given radiotherapy in the inner breast area (3). IMLN radiotherapy is strongly recommended for breast cancer with an ALN status of N1a or N2a (3). Therefore, IMLN biopsy in such patients has little significance for the decision-making of a follow-up treatment plan. Thus, for ethical reasons, all patients enrolled in this study were clinically ALN-negative patients.

By analyzing the data of 179 patients with primary breast cancer after neoadjuvant therapy, Bi et al. (28) found that after neoadjuvant therapy, the imaging rate of IM-SLN was 31.8% (57/179), the metastasis rate of IM-SLN was 7.1% (4/56), and ALN metastasis was associated with them. It is considered that IM-SLNB can further improve the definition of lymph node pCR after neoadjuvant therapy.

Nevertheless, traditional tracer methods are inefficient in IM-SLNB. The retrospective analysis by Manca et al. (17) showed that the detection rate of IM-SLN using a traditional radiotracer injection technique was 13%-37%, while other studies reported lower rates of 11%-16% (29, 30). In addition, the rate of IMLN metastasis is relatively low. Thus, although the AJCC adopted the concept of IM-SLNB as early as in 2009, the surgical method of IMLN has basically not changed, and the clinical application of IM-SLNB is limited.

The emergence of improved injection techniques has greatly improved the clinical feasibility of IM-SLNB. In a controlled study of 470 patients, the detection rate of IM-SLN in the modified injection group was significantly higher than in the traditional method group (71.1% vs. 15.5%, P<0.001) (22). In this study, ^99m^Tc-Rituximab was selected as the tracer (13). It was injected using either the traditional peritumoral subcutaneous injection method or the two- or four-site intraglandular injection method. After using the improved injection method, the total detection rate of ^99m^Tc-rituximab-guided IM-SLN was 58.8% (47/80), significantly higher than the traditional method (9.4%). Some studies have shown that with the improved injection method, the detection rate of IM-SLN could reach 63.3%-75% (31, 32). The rates in the present study were slightly lower than in the previous studies, which could be influenced by the tracer used (^99m^Tc-SC) and the injection scheme. Whether there are differences in the detection rate of IMLN among different tracers needs to be further explored in future studies. Another technical problem that might affect the detection rate of IM-SLN is ensuring the injection of the radioactive tracer into the gland. Intraglandular injection requires extensive experience, and it is often difficult to inject radioactive tracers to the correct depth in the gland. Even under ultrasound guidance, the accuracy of the injection depth cannot be guaranteed, especially in the learning stage of the method, and it could have lowered the detection rate.

Mudun et al. (8) compared the effects of tracer injection in different parts on the detection rate of SLN and found that the detection rate of IMLN using four injection sites around the tumor (22.2%) was better than using a single site (8.4%). Therefore, two and four sites were used in this study, but the results showed no significant differences in the detection rate between the two- and four-site methods. Nevertheless, the detection rate of the two-site method reached 61.7%, higher (but not statistically significant) than 50% of the four-site method. It could be hypothesized that injecting at 3 and 9 o’clock results in at least one injection being close to the IMLN; therefore, adding the 6 and 12 o’clock positions does not improve IMLN detection. In the four-site group, the 9 o’clock point in the left breast and the 3 o’clock point in the right breast are relatively close to the IMLNs, resulting in a large amount of residual radioactivity at the injection sites. The radioactivity from IM-SLN might be covered up during the surgical exploration, affecting the detection of IM-SLNs. Hence, using only two sites would be enough, consume less isotope, be less expensive, and expose the patients to a smaller radiation dose.

In this study, there were no adverse events, except for some patients who felt pain during the injections. In fact, due to the limited dosage of tracer, very few adverse events caused by ^99m^Tc-rituximab have been reported in the literature, irrespective of the disease (13, 33–35). Still, the sample size was probably too small to observe rare events. Large-scale studies will be necessary to confirm the safety of ^99m^Tc-rituximab.

This study has limitations. The injection volume of the traditional methods was small (0.2-0.5 ml/injection site), and there was no small-volume injection control group in this study. The patients were clinically negative for ALNs and might be at low risk of IMLN metastasis. The IM-SLN visualization was designed to find the SLNs, independent of whether the lymph nodes examined were metastatic or not. Therefore, the pathological diagnosis of IM-SLN was not analyzed. Finally, the group assignment was not randomized and was left to the patients’ choice. Although the general characteristics of patients were the same, more patients selected the two-site group because of the fear of greater injection pain in the four-site group. Future studies should focus on the IMLN detection methods and rates in different populations of patients.

In conclusion, ^99m^Tc-rituximab radiotracer injected using the two- or four-site method might achieve higher IM-SLN detection rates and comparable detection rates of A-SLNs compared with the peritumoral method. This study suggests methods for an improved detection rate of IM-SLN, which will highlight the value of IM-SLNB in guiding subsequent treatment. This method will also assist in determining the clinical staging of breast cancer patients using minimally invasive surgery.

## Data availability statement

The original contributions presented in the study are included in the article/supplementary material. Further inquiries can be directed to the corresponding authors.

## Ethics statement

The studies involving human participants were reviewed and approved by the ethics committee of Shengli Clinical Medical College of Fujian Medical University, Fujian Provincial Hospital. The patients/participants provided their written informed consent to participate in this study.

## Author contributions

WC: Founding acquisition, Resource, Supervision, Writing-review& editing. YS: Writing, Visualization, Methodology, Data curation. HZ: Resource, Methodology. YZ: Data curation, Formal analysis. LZ: Methodology. ML: Resources, Validation. ZL: Visualization, Methodology. MY, SY, and YMZ: Data curation. All authors contributed to the article and approved the submitted version.
